# Supplementary material for: Realisation of de Gennes’ absolute superconducting switch with a heavy metal interface
Source: Nat Commun. 2025 Jul 1;16:5674. doi: 10.1038/s41467-025-61267-2 (PMC12218286; doi:10.1038/s41467-025-61267-2)
Supplement: Supplementary file 1 — Supplementary Information [file 41467_2025_61267_MOESM1_ESM.pdf]

Supplementary Information

Realisation of de Gennes' Absolute Superconducting Switch with a Heavy Metal Interface

Hisakazu Matsuki[1], Alberto Hijano[2,3,4], Grzegorz P. Mazur[1,5], Stefan Ilić[2,4], Binbin Wang[6], Iuliia Alekhina[1], Kohei Ohnishi[7], Sachio Komori[8], Yang Li[1,9], Nadia Stelmashenko[1], Niladri Banerjee[10], Lesley F. Cohen[10], David W. McComb[6], F. Sebastián Bergeret[2,11], Guang Yang[1,12,13*] and Jason W. A. Robinson[1*]

1. Department of Materials Science & Metallurgy, University of Cambridge, 27 Charles Babbage Road, Cambridge CB3 0FS, U.K.
2. Centro de Física de Materiales (CFM-MPC) Centro Mixto CSIC-UPV/EHU, E-20018 Donostia-San Sebastián, Spain
3. Department of Condensed Matter Physics, University of the Basque Country UPV/EHU, 48080 Bilbao, Spain
4. Department of Physics and Nanoscience Center, University of Jyväskylä, P.O. Box 35 (YFL), Jyväskylä, FI-40014 Finland
5. QuTech and Kavli Institute of NanoScience, Delft University of Technology, 2600 GA Delft, The Netherlands
6. Department of Materials Science and Engineering, The Ohio State University, Columbus, OH 43210, USA.
7. Department of Electrical, Electronic and Communication Engineering, Kindai University, Osaka 577-8502, Japan
8. Department of Physics, Nagoya University, Nagoya 464-8602, Japan
9. Cambridge Graphene Centre, University of Cambridge, 9 JJ Thomson Avenue, Cambridge CB3 0FA, U.K.
10. Department of Physics, Blackett Laboratory, Imperial College London, London SW7 2AZ, U.K.
11. Donostia International Physics Center (DIPC), 20018 Donostia–San Sebastián, Spain
12. National Key Laboratory of Spintronics, Hangzhou International Innovation Institute, Beihang University, Hangzhou 311115, China
13. School of Integrated Circuit Science and Engineering, Beihang University, Beijing 100191, China

*e-mail: gy251@buaa.edu.cn, jjr33@cam.ac.uk

Supplementary Note 1. Extended Data of the Absolute Superconducting Switch

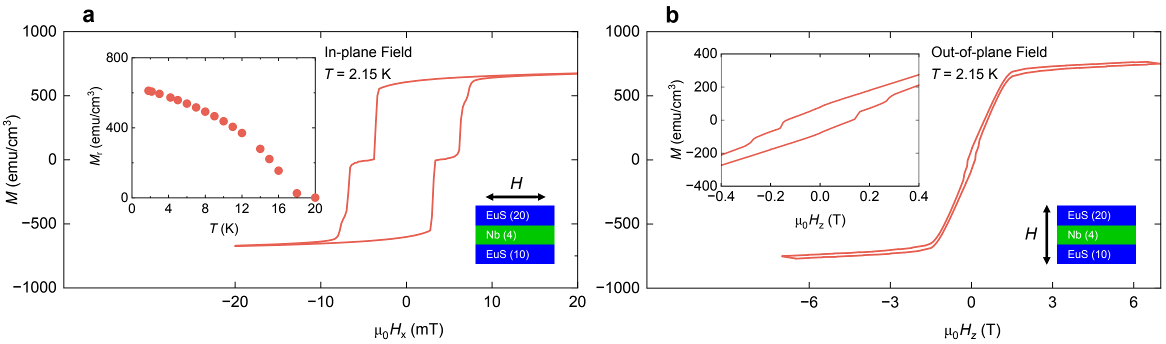

Supplementary Figure 1: In-plane (a) and out-of-plane (b) magnetic hysteresis loops of NbO_x(3 nm)/EuS (20 nm)/Nb(4 nm)/EuS (10 nm)/SiO_2//Si structure at 2.15 K. Inset of **b** is the same out-of-plane $M(H)$ hysteresis loop with a smaller field range.

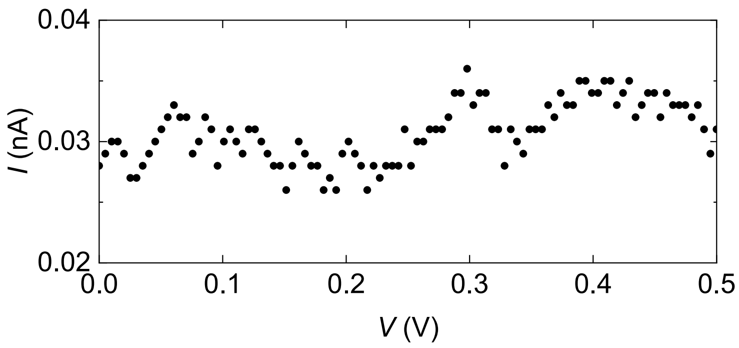

Supplementary Figure 2: The current (*I*) - voltage (*V*) characteristics of an uncapped 30-nm-thick EuS thin-film measured at room temperature. The contact resistance is larger than 10 GΩ ($\rho > 3 \times 10^4$ Ω·cm). In EuS/Nb heterostructures, all current passes through the metallic layer.

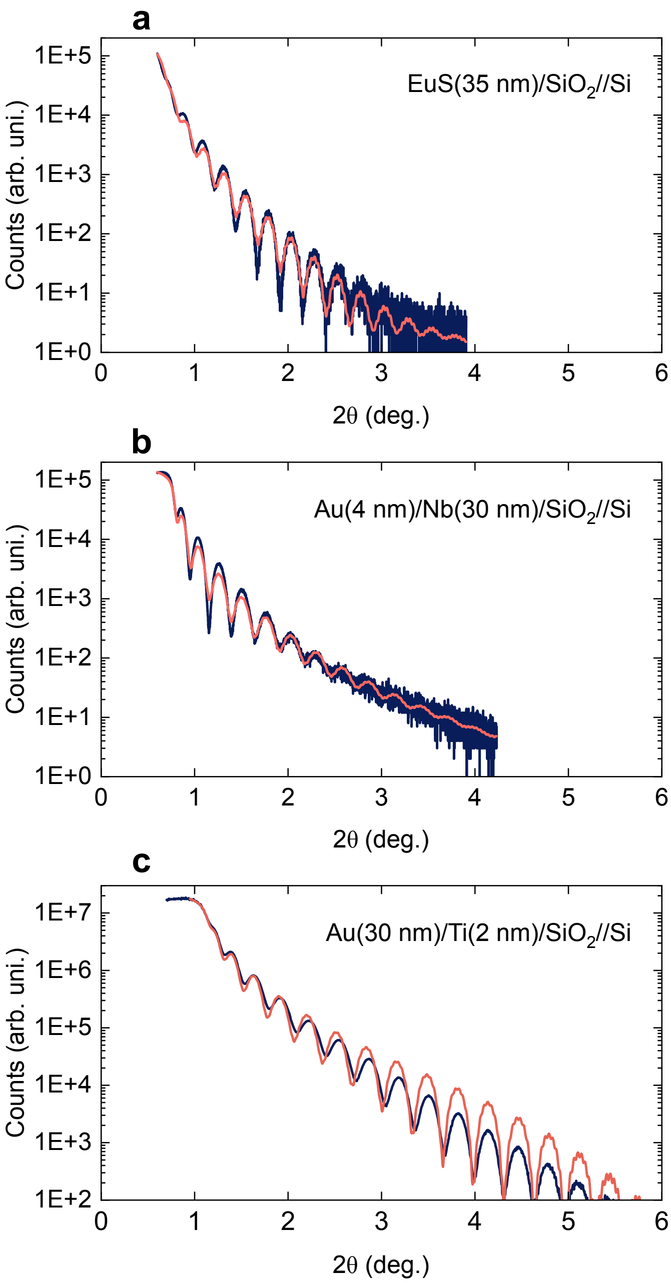

Supplementary Figure 3: The X-ray reflectivity measurements. Top:35-nm-thick EuS without capping, middle: 30-nm-thick Nb capped by a 4-nm-thick Au layer, and bottom: 30-nm-thick Au with 2-nm-thick Ti seed layer below. Dark blue curves are the measurement data and red curves are the fitting curves.

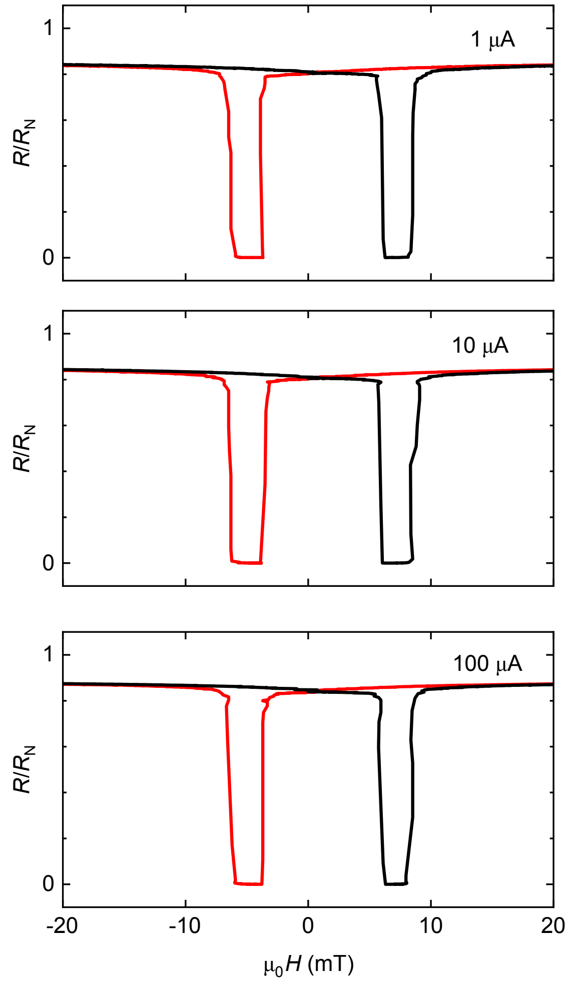

Supplementary Figure 4: *R*(*H*) of an unpatterned NbO_x(3 nm)/EuS(20 nm)/Nb(4 nm)/EuS(10 nm)/SiO_2//Si (Device 3) structure at 2.15 K with different current bias. (From top: *I* = 1 µA, 10 µA, 100 µA). There is no significant effect on *R*(*H*). Red (black) curves indicate a decreasing (increasing) in-plane magnetic field.

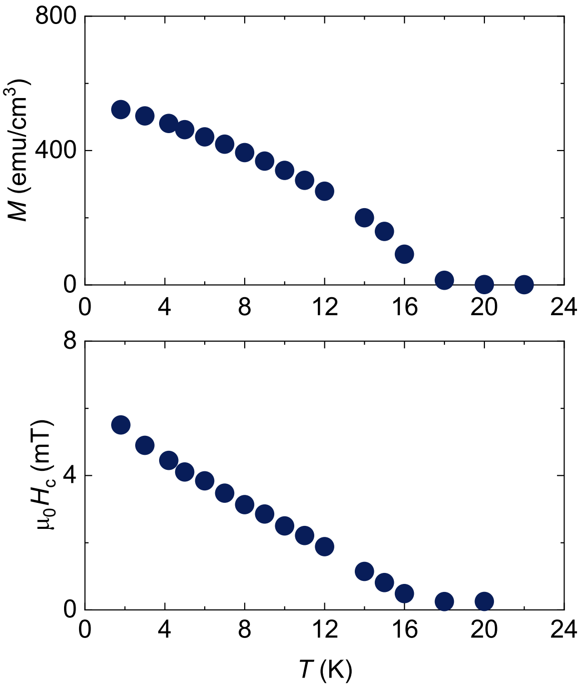

Supplementary Figure 5: Remanence (top) and coercive field (bottom) of a 30-nm-thick EuS thin-film as a function of temperature extracted from individual *M*(*H*) loops measured at different *T*. Curie temperature is close to the theoretical value of bulk EuS of 16.6 K.

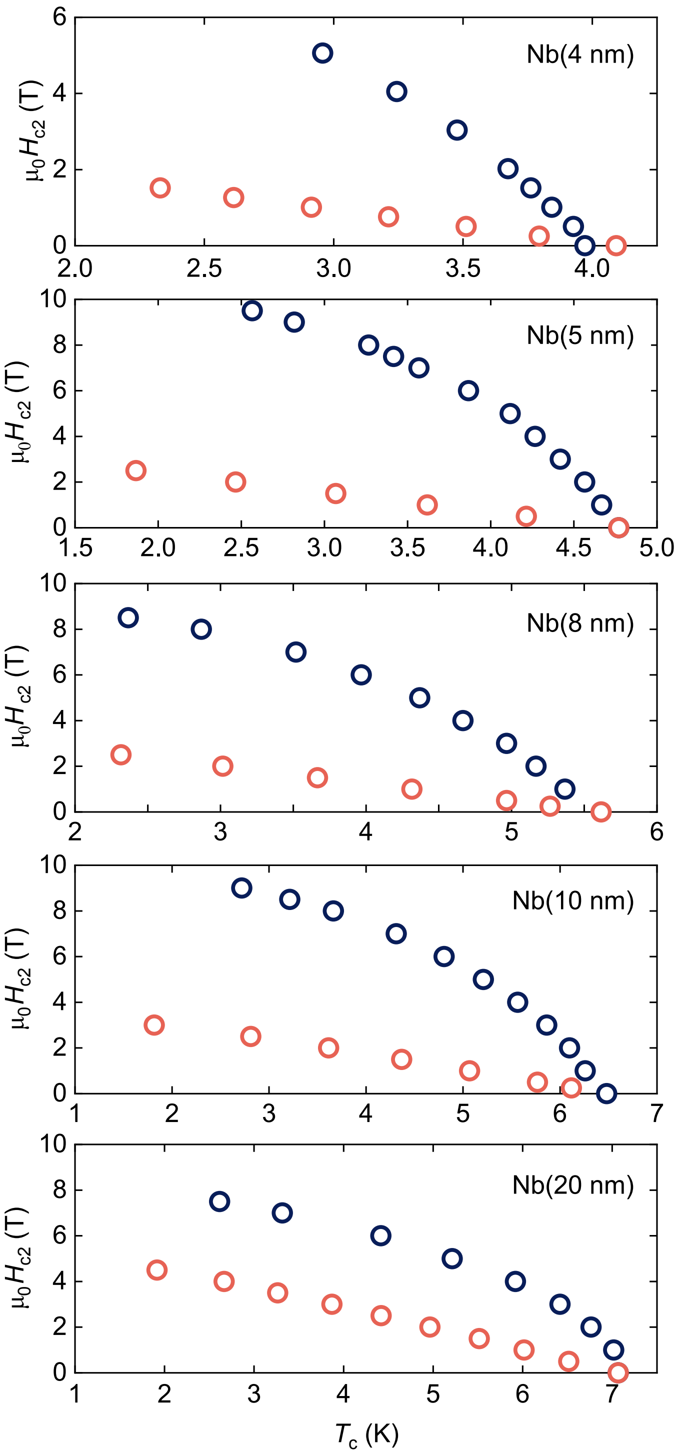

Supplementary Figure 6: In-plane (in dark blue) and out-of-plane (in red) critical fields of NbO_x(3 nm) /EuS(30 nm)/Nb(d_{Nb} nm) structures without showing an infinite magnetoresistance. Nb thicknesses are annotated in the figures. Dirty-limit coherence length (ξ_s) of Nb is calculated from the dependence of critical temperature in out-of-plane magnetic fields of the NbO_x(3 nm)/EuS(30 nm)/Nb(20 nm)/SiO_2//Si structure using the relation of $\xi_{GL}(0) = [-(dH_{c2}(T)/dT)(2\pi T_{c0}/\Phi_0)]^{-1/2}$, and $\xi_s = \frac{2}{\pi}\xi_{GL}(0)$, where T_{c0} is the critical temperature at zero magnetic field, Φ_0 is the flux quantum, and $\xi_{GL}(0)$ is the zero-temperature Ginzburg-Landau coherence length. ξ_s of the 20-nm-thick Nb is 4.6 nm.

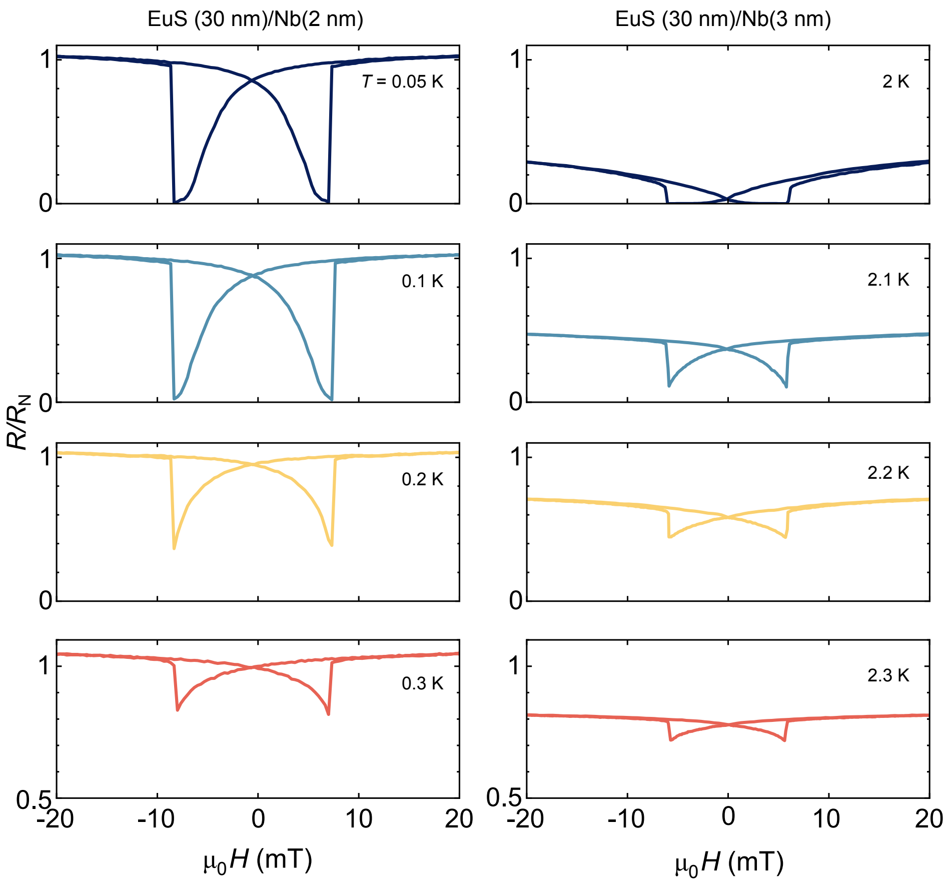

Supplementary Figure 7: Extended data of the normalised *R*(*H*) traces of NbO_x(3 nm)/EuS(30 nm)/Nb(2 nm)/SiO_2//Si and NbO_x(3 nm)/EuS(30 nm)/Nb(3 nm)/SiO_2//Si devices across their superconducting transitions.

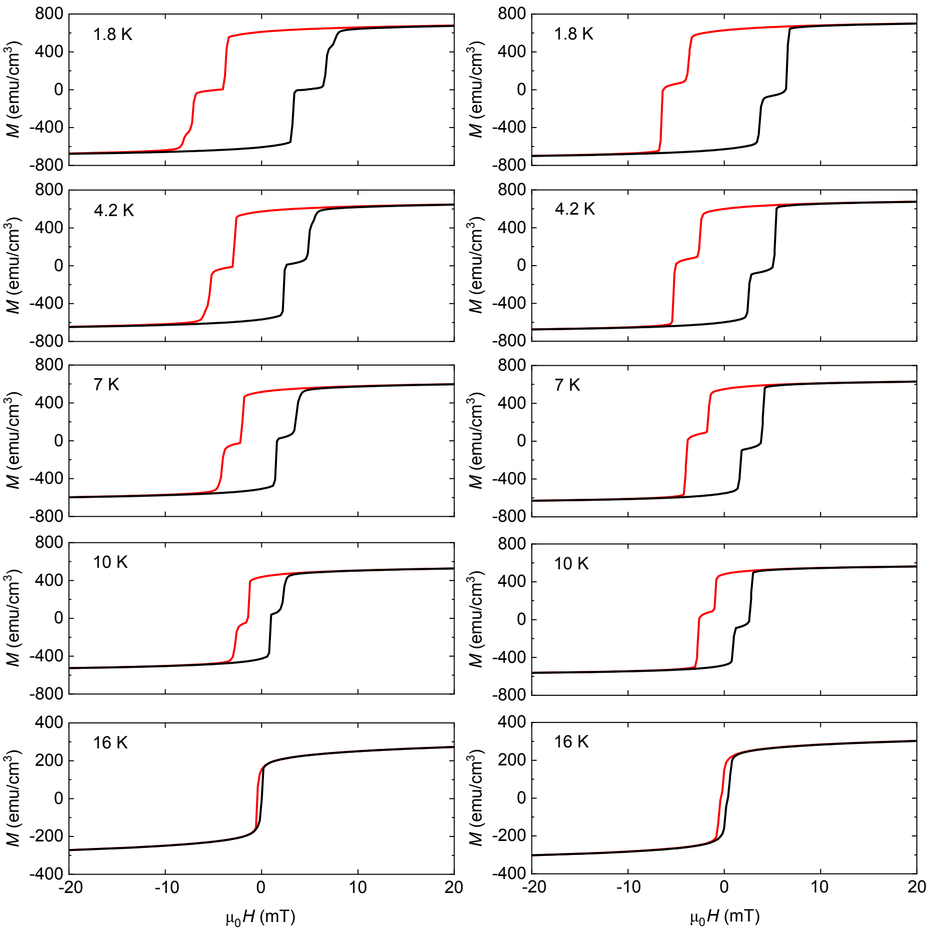

Supplementary Figure 8: M(H) hysteresis loops of NbO_x(3 nm)/EuS(20 nm)/Nb(4 nm)/EuS(10 nm) /SiO_2//Si (Device 1, left column), and NbO_x(3 nm)/EuS(20 nm)/Au(20 nm)/Nb(4 nm)/EuS(10 nm)/SiO_2//Si (Device 2, right column). Red (black) curves indicate a decreasing (increasing) in-plane magnetic field.

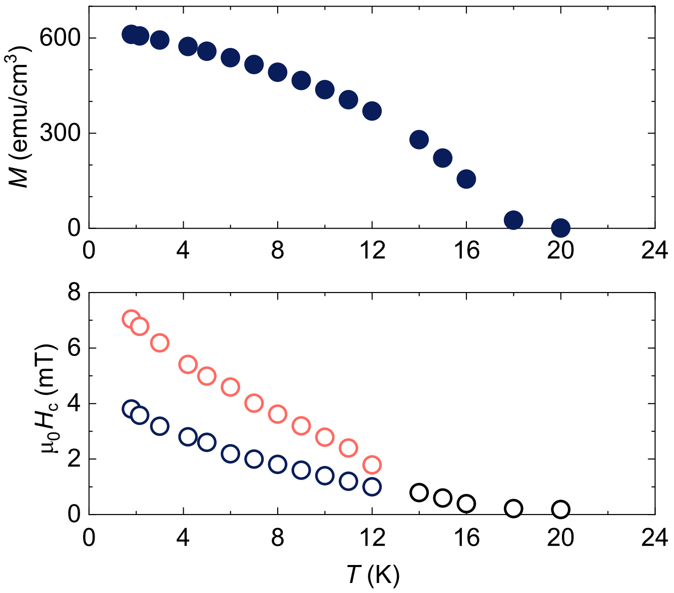

Supplementary Figure 9: Remanence (top) and coercive fields (bottom) of a NbO_x(3 nm)/EuS(20 nm) /Nb(4 nm)/EuS(10 nm)/SiO_2//Si structure. Two distinctive switching steps in the M(H) traces corresponds to the coercive fields of two EuS layers with different thicknesses (in red and blue) is extracted at temperatures below 12 K. At above 12 K, two transition steps in M(H) traces merge into one (in black).

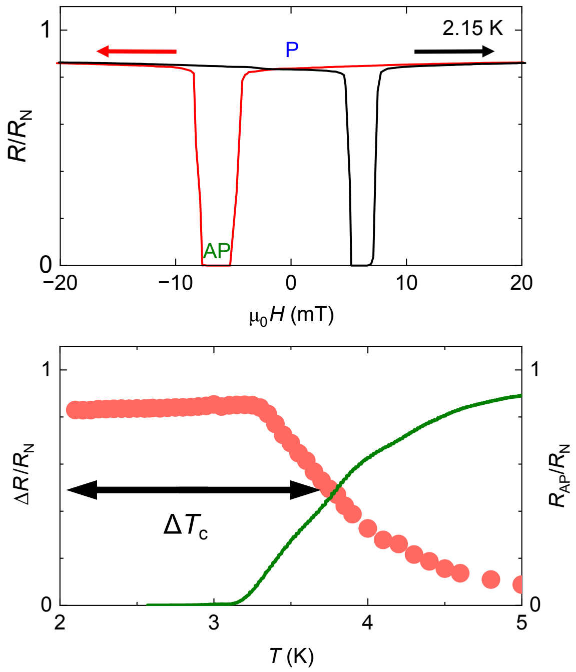

Supplementary Figure 10: Superconducting switch performance of an unpatterned NbO_x(3 nm) /EuS(20 nm)/Nb(4 nm)/EuS(10 nm)/SiO_2//Si structure (Noted as Device 3) grown in the same condition as the device shown in Fig. 2a and b. Top: $R(H)$ at 2.15 K. Red (black) curves indicate a decreasing (increasing) in-plane magnetic field. Bottom: Normalised $R_{AP}(T)/R_N(T)$ (green line, right axis) and $\Delta R(T)/R_N(T)$ of individual $R(H)$ scans (in pink, left axis). $\Delta T_c/T_{c,AP}$ reaches 50 %.

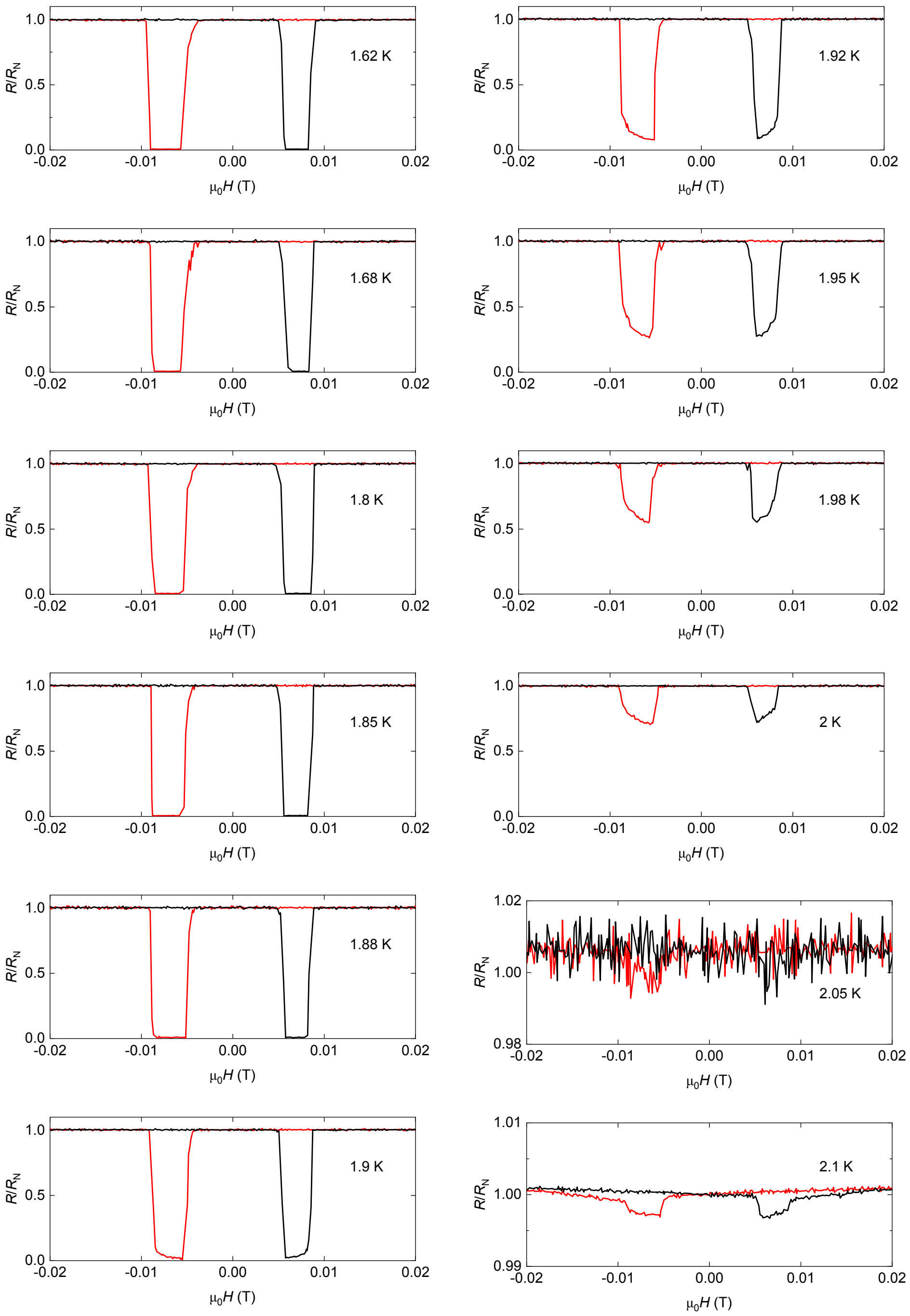

Supplementary Figure 11. Extended data of normalised $R(H)$ scans of an unpatterned NbO_x(3 nm)/ EuS(20 nm)/Au(20 nm)/Nb(4 nm)/EuS(10 nm)/SiO_2//Si structure at temperatures across T_c. Red (black) curves indicate a decreasing (increasing) in-plane magnetic field.

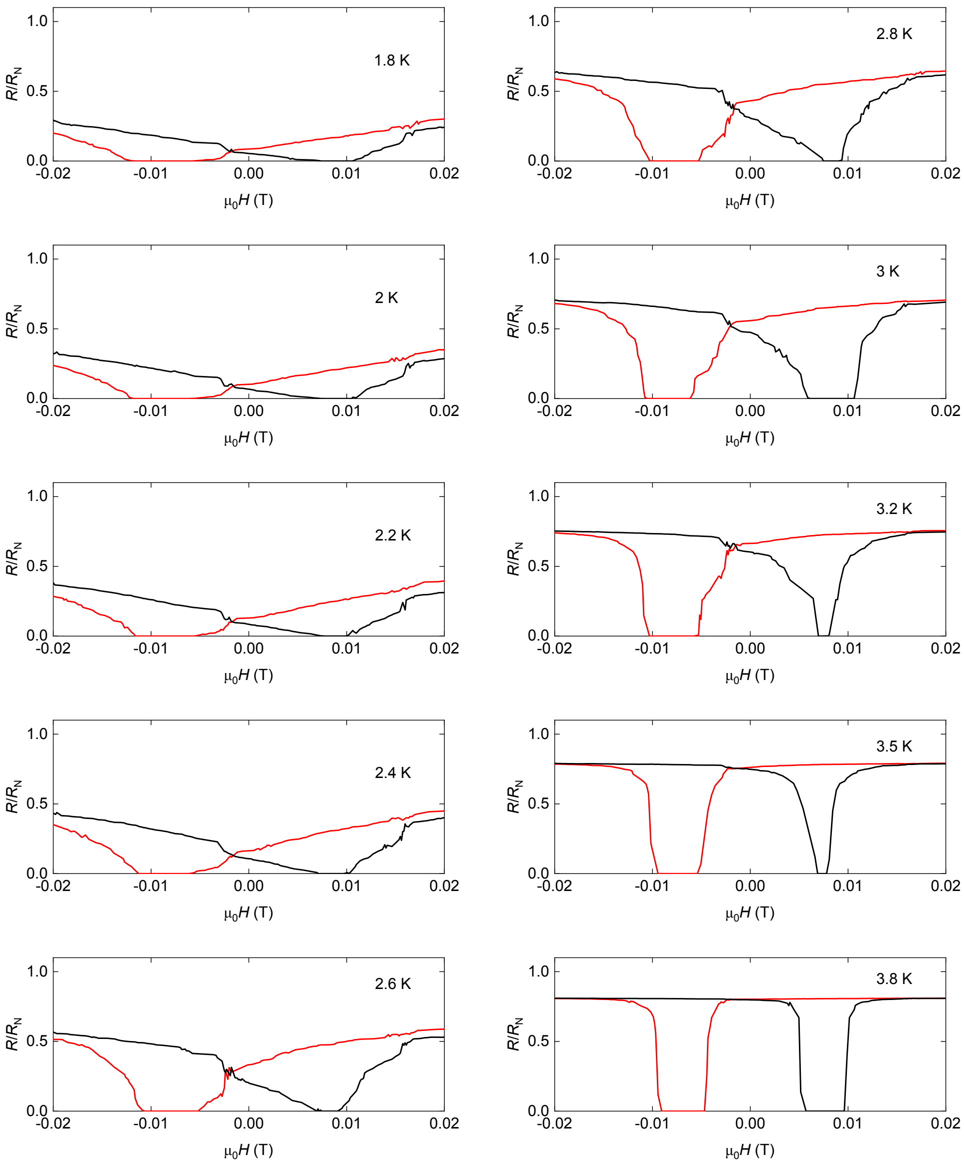
1.8 K
2 K
2.2 K
2.4 K
2.6 K
2.8 K
3 K
3.2 K
3.5 K
3.8 K
R/R_N
μ0H (T)

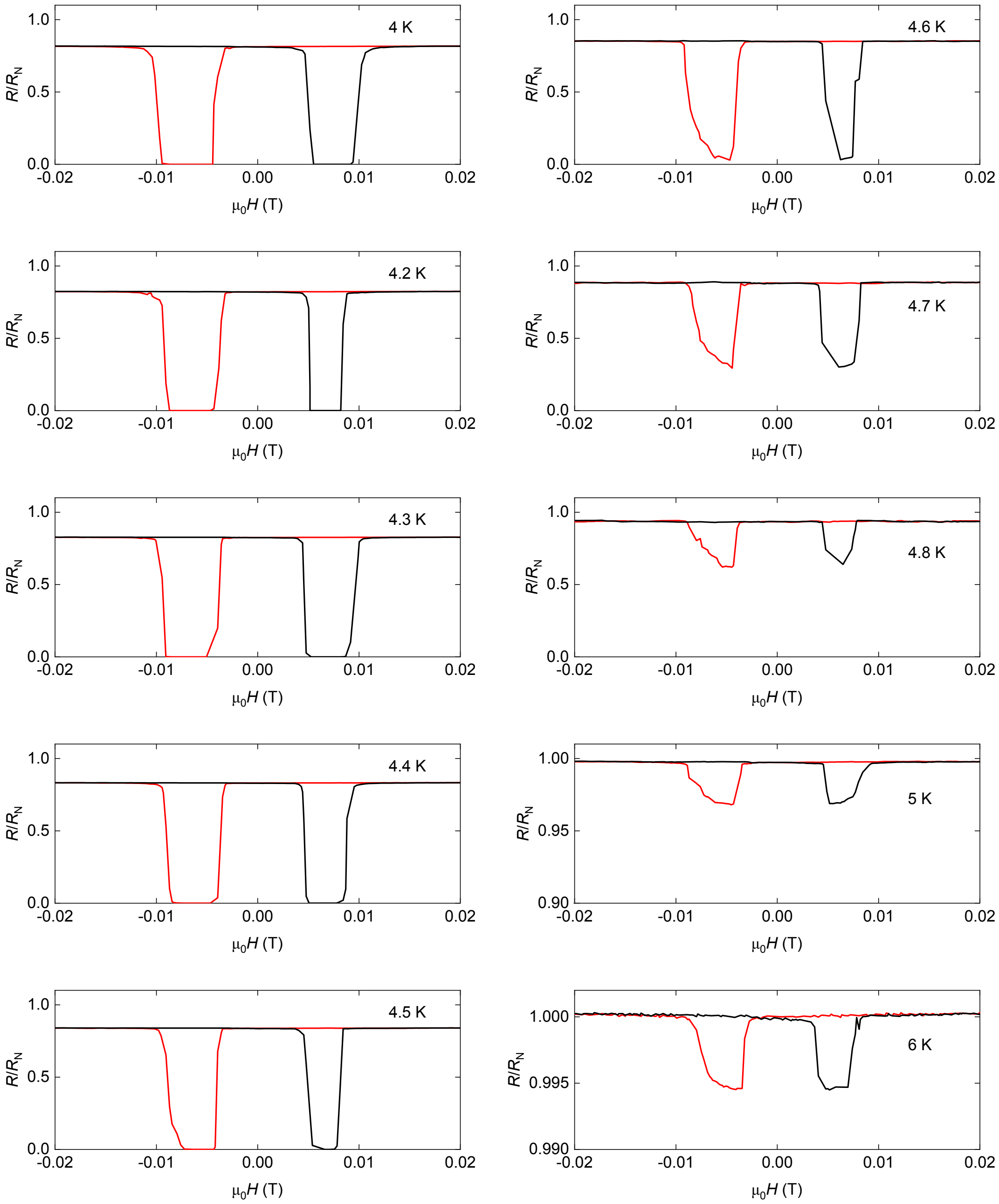

Supplementary Figure 12. Extended data of normalised *R*(*H*) scans of an unpatterned NbO_x(3 nm)/EuS(20 nm)/Nb(4 nm)/EuS(10 nm)/SiO_2//Si structure at temperatures across T_c. Red (black) curves indicate a decreasing (increasing) in-plane magnetic field.

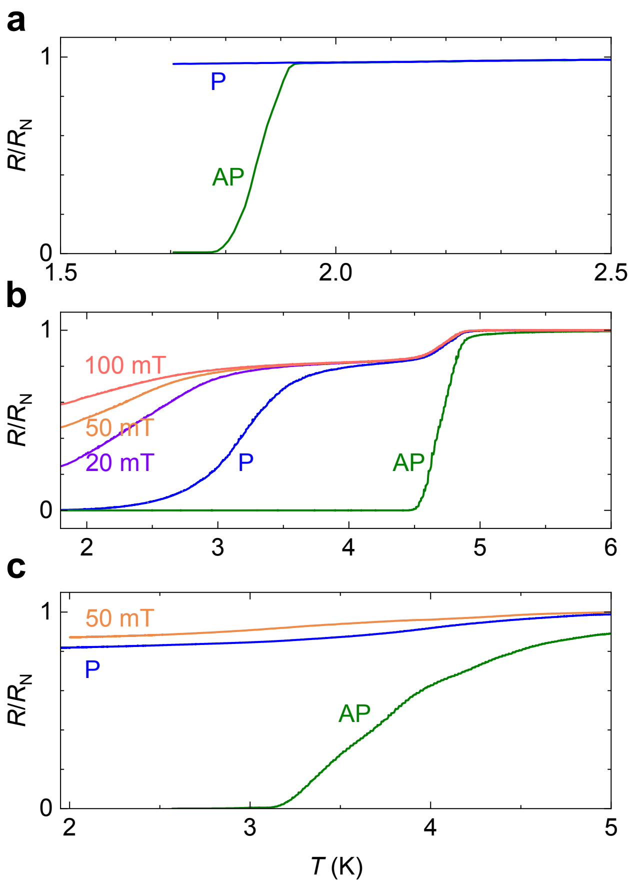

Supplementary Figure 13: The temperature dependence of the normalised resistance in the P-, AP-states, and applying in-plane magnetic fields of different switches. **a** NbO_x(3 nm)/EuS(20 nm)/Au(20 nm)/ Nb(4 nm)/EuS(10 nm)/SiO_2//Si (Device 2). **b** NbO_x(3 nm)/EuS(20 nm)/Nb(4 nm)/EuS(10 nm)/SiO_2//Si (Device 1). **c** NbO_x(3 nm)/EuS(20 nm)/Nb(4 nm)/EuS(10 nm)/SiO_2//Si (Device 3). The zero-field T-dependence of R_P and R_{AP} are determined using the following measurement sequence: ***R***$\boldsymbol{R_P(T)}$ **trace:** 1: the structure is warmed to the normal state; 2: an in-plane magnetic field of +20 mT is applied to set the P state; 3: the field is then removed so $R_P(T)$ can then be measured in zero-field cooling. $\boldsymbol{R_{AP}(T)}$ **trace:** 1: the device is cooled to the superconducting transition temperature; 2: an in-plane magnetic field of +20 mT is applied to set the P state; 3: an in-plane magnetic field of -4 mT is applied to set the AP state, where the switch is in the zero-resistance state; 4: the field is then removed and the device is warmed to the normal state while maintaining the AP state; 5: $R_{AP}(T)$ is then be measured in zero-field cooling. The other curves are measured in field-cooling with corresponding in-plane magnetic fields. We obtain a superconducting switch efficiency of about $\Delta T_c/T_{c,AP}$ = 30 % in **b**, and larger than 50 % in **c**.

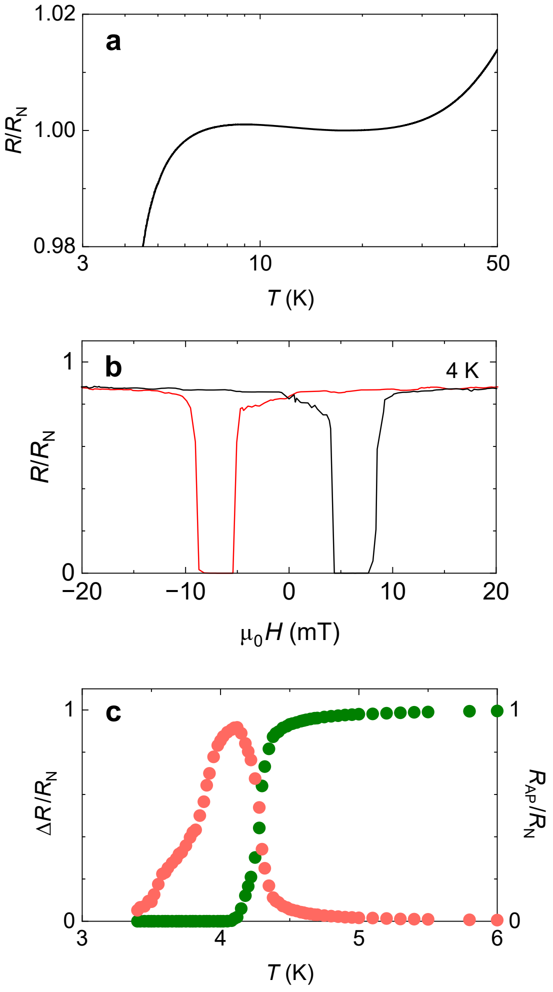

Supplementary Figure 14. Electrical characterisations of NbO_x(3 nm)/EuS(20 nm)/Nb(3 nm)/ EuS(10 nm)/SiO_2//Si device (Device 4). a: $R(T)$ in zero-field cooling. **b**: $R(H)$ at 4 K. **c**: Superconducting switch performance. $R_{AP}(T)/R_N(T)$ (in green) and $\Delta R(T)/R_N(T)$ of individual $R(H)$ scans (in pink).

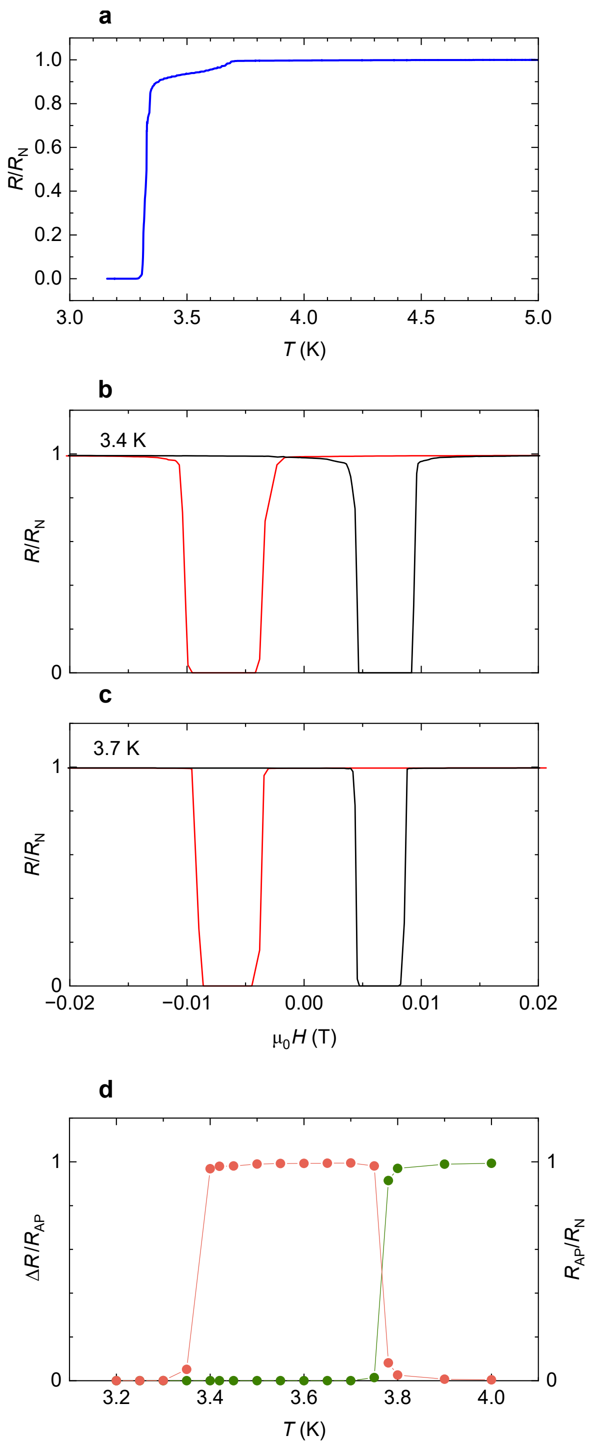

Supplementary Figure 15: Electrical characterisations of NbO_x(3 nm)/EuS(20 nm)/Al(8 nm)/Nb(4 nm)/EuS(10 nm)/SiO_2//Si device. a $R(T)$ in zero-field cooling after magnetising the EuS at 6 K. **b**, **c** $R(H)$ at 3.4 K and 3.7 K, respectively. **d**: superconducting switch performance. $R_{AP}(T)/R_N(T)$ (in green) and $\Delta R(T)/R_N(T)$ (in pink) of individual $R(H)$ scans.

Supplementary Note 2. Computing the Critical Temperature of FI/N/S/FI Heterostructures using Quasiclassical Green's Functions

We study the critical temperature (T_c) of the spin-switch using the quasiclassical Green's function (GF) technique[1-6]. In diffusive systems, the quasiclassical GF $\breve{g}$ is determined by a diffusion-like equation known as Usadel equation[3]. Together with the normalisation condition $\breve{g}^2 = \breve{1}$ and the boundary conditions describing the hybrid interfaces determine the value of $\breve{g}$, from which the properties of the system, such as the critical temperature, may be extracted.

Assuming that the thicknesses of the layers are much smaller than the coherence length, the GF in the S and N layers can be assumed to be constant, so that the Usadel equation may be integrated over the thickness of the layers. Using the Kuprianov-Lukichev boundary condition[7] to describe the S/N interface, the Usadel equations describing the S and N layers become

$$\left[(\omega + ih\sigma_3)\tau_3 + \Delta\tau_1 + \frac{\sigma_i \breve{g}_\mathrm{S} \sigma_i}{8\tau_\mathrm{S}^\mathrm{so}} + \Gamma_\mathrm{S}\breve{g}_\mathrm{N}, \breve{g}_\mathrm{S}\right] = 0, \tag{1a}$$

$$\left[(\omega + ih\sigma_3)\tau_3 + \frac{\sigma_i \breve{g}_\mathrm{N} \sigma_i}{8\tau_\mathrm{N}^\mathrm{so}} + \Gamma_\mathrm{N}\breve{g}_\mathrm{S}, \breve{g}_\mathrm{N}\right] = 0, \tag{1b}$$

where, $\omega = 2\pi T(n + 1/2)$ with $n \in \mathbb{Z}$ is the Matsubara frequency, Δ is the superconducting order parameter, and $\tau_{\mathrm{so,S/N}}$ are the spin-orbit scattering times of the S and N layers, introduced by impurities with spin-orbit coupling. $h_{\mathrm{S/N}}$ are the effective exchange fields introduced by the FI layers on the S and N layers. Assuming that the thicknesses of the layers are much smaller than the coherence length, the exchange field may be taken to be homogeneous over each layer, the effective exchange field being inversely proportional to the thickness of the layer $h_{\mathrm{S/N}}(d) = \kappa_{\mathrm{int,S/N}}/d_{\mathrm{S/N}}$[1-4], where κ_{int} is a parameter quantifying the interfacial exchange field at the FI/metal interfaces with dimensions of energy times length.

Because we are dealing with superconductivity and spin-independent fields, the GFs on the N and S layers are 4×4 matrices in Nambu-spin space. The matrices σ_i and τ_i $(i = 1, 2, 3)$ in Supplementary Eq. (1) are the Pauli matrices in spin and Nambu space, respectively. Summation over repeated indices is implied. The coupling of the N and S layers is determined by the effective rates[61]

$$\Gamma_\mathrm{S} = \frac{v_\mathrm{S}}{2\pi d_\mathrm{S}\rho_\mathrm{int}}, \Gamma_\mathrm{N} = \frac{v_\mathrm{S}^2}{2\pi v_\mathrm{N} d_\mathrm{N}\rho_\mathrm{int}}, \tag{2}$$

with $v_{\mathrm{S/N}}$ the Fermi velocities and ρ_{int} is a dimensionless parameter describing the resistance of the S/N interface, with $\rho_{\mathrm{int}} = \infty$ corresponding to a completely opaque interface. Γ_N and Γ_S describe the proximity effect and its inverse, respectively.

Close to T_c, the GF may be linearised with respect to Δ as $\breve{g} = \mathrm{sgn}(\omega)\tau_3 + \hat{f}\tau_1$, where $\hat{f} = O(\Delta)$ is the anomalous part of the GF, describing the superconducting correlations. The exchange fields introduced by the exchange interaction with the FI layers are either in the parallel or antiparallel configurations, so without any loss of generality we assume that they lie along the *z*-axis. In this case, the anomalous GF will contain a

singlet and a z-triplet projection: $\hat{f}_{S/N} = f_{S/N,0}\sigma_0 + f_{S/N,3}\sigma_3$, with f_0 describing the singlet correlations and f_3 the triplet correlations. Solving the equation system Supplementary Eq. (1), we obtain the value of the GF at the S and the N layers. The singlet part in the superconducting layer, from which T_c is determined, takes the form

$$f_{\mathrm{S},0} = \Delta \frac{(h_{\mathrm{N}}^2 + \Omega_{\mathrm{N},0}\Omega_{\mathrm{N},3})\Omega_{\mathrm{S},3} - \Gamma_{\mathrm{S}}\Gamma_{\mathrm{N}}\Omega_{\mathrm{N},0}}{\Gamma_{\mathrm{S}}^2\Gamma_{\mathrm{N}}^2 + \Gamma_{\mathrm{S}}\Gamma_{\mathrm{N}}(2h_{\mathrm{S}}h_{\mathrm{N}} - \Omega_{\mathrm{S},0}\Omega_{\mathrm{N},0} - \Omega_{\mathrm{S},3}\Omega_{\mathrm{N},3}) + (h_{\mathrm{S}}^2 + \Omega_{\mathrm{S},0}\Omega_{\mathrm{S},3})(h_{\mathrm{N}}^2 + \Omega_{\mathrm{N},0}\Omega_{\mathrm{N},3})}, \quad (3)$$

where, $\Omega_{\mathrm{S/N},0} = |\omega| + \Gamma_{\mathrm{S/N}}$ and $\Omega_{\mathrm{S/N},3} = |\omega| + \Gamma_{\mathrm{S/N}} + 1/(2\tau_{\mathrm{S/N}}^{\mathrm{so}})$.

The critical temperature of the bilayer T_c is given by the self-consistency equation[9]

$$\ln\left(\frac{T_{\mathrm{c}}}{T_{\mathrm{c}}^{\mathrm{BCS}}}\right) = 2\pi T_{\mathrm{c}} \sum_{\omega>0} \left[\frac{f_{\mathrm{S},0}}{\Delta} - \frac{1}{\omega}\right], \quad (4)$$

where $T_{\mathrm{c}}^{\mathrm{BCS}}$ is the critical temperature of the bulk superconductor. Inserting Supplementary Eq. (3) into Supplementary Eq. (4) and solving T_c, we obtain the critical temperature of the bilayer.

We have compared the introduced theoretical model to experimental data to explain the enhancement in the superconducting switch efficiency observed in samples with Au interlayer. We first perform a fitting of the parameters of the model, we consider an Au/Nb bilayer with no EuS layers, i.e. no exchange field $h_{\mathrm{S}} = h_{\mathrm{N}} = 0$. The thickness of Nb of the samples studied was d_{Nb} = 4 nm, while the thickness of the Au layer laid in the $d_{\mathrm{Au}} \in [0, 20]$ nm range. T_c of Au(d_{Au})/Nb(4 nm)/SiO_2//Si bilayers and its theoretical model is shown in **Supplementary Fig. 17**.

The following values for the Au and Nb Fermi velocities $v_{Nb} = v_{Au} = 3 \times 10^5$ $m \cdot s^{-1}$ [10], and the spin-orbit relaxation times τ_{Nb}^{so} ~ 6 meV^{-1} [11] and τ_{Au}^{so} ~ 2.4 meV^{-1} [12] were used for the fitting. The Au/Nb interface resistance was extracted from the critical temperature dependence on d_{Au} in **Supplementary Fig. 17**, ρ_{int} ~ 20. Next, we consider the EuS/Au/Nb/EuS structure to fit the exchange interaction at the EuS/Nb and EuS/Au interfaces. Appropriate parameters reproducing the enhancement of the efficiency $\Delta T_c/T_{c,AP}$ and absolute switching for thick Au layers $\Delta T_c/T_{c,AP}$ ~ 1 (see Fig. 4**a** and **b**) are $\kappa_{EuS/Nb}$ ~ 1.2 meV·nm and $\kappa_{EuS/Au}$ ~ 1.5 meV·nm. The superconducting switch efficiency of EuS/Au(d_{Au})/Nb(4 nm)/EuS device with different $\kappa_{EuS/Au}$ is presented in **Supplementary Fig. 16.**

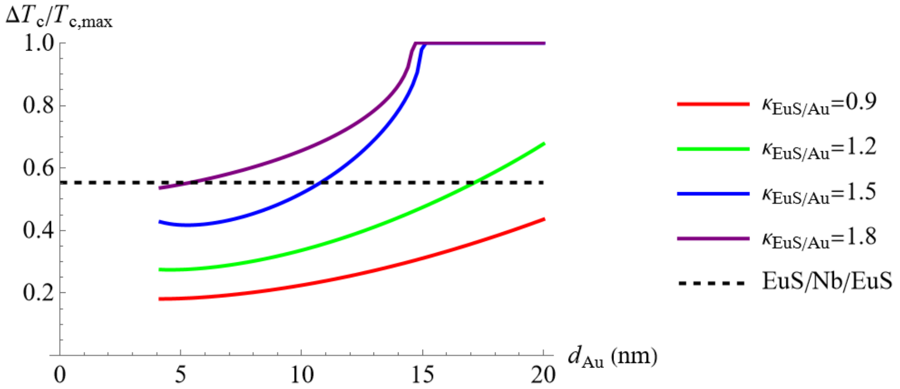

Supplementary Figure 16: Superconducting switch efficiency of EuS/Au(d_{Au})/Nb(4 nm)/EuS switch with modified exchange field at EuS/Au interface. For $\kappa_{EuS/Au}$ = 1.2 meV·nm, the device achieves the same superconducting switch efficiency as a device without Au insertion at d_{Au} ~ 17 nm, though with a reduced $T_{c,AP}$. For $\kappa_{EuS/Au}$ = 1.5 meV·nm, an absolute superconducting switch is achieved with d_{Au} > 15 nm.

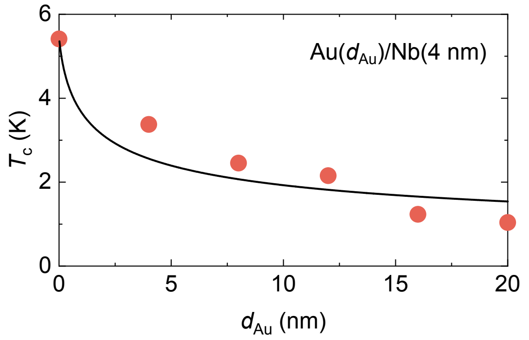

Supplementary Figure 17: T_c of Au(d_{Au})/Nb(4 nm)/SiO_2//Si bilayers. Nb thickness is fixed to 4 nm. The red data points correspond to the experimental measurements of T_c, and the black line is the theoretical model. T_c of 4-nm-thick Nb with d_{Au} = 0 nm is obtained from the T_c of a 4-nm-thick Nb capped by a 2-nm-thick MgO layer.

References:

1. Eilenberger, G. Transformation of Gorkov's equation for type II superconductors into transport-like equations. *Z. Für Phys. Hadrons Nucl.* **214**, 195–213 (1968).
2. Larkin, A. & Ovchinnikov, Y. N. Quasiclassical method in the theory of superconductivity. *Zh Eksp Teor Fiz* **55**, (1969).
3. Usadel, K. D. Generalized diffusion equation for superconducting alloys. *Phys. Rev. Lett.* **25**, 507–509 (1970).
4. Volkov, A. F., Zaitsev, A. V. & Klapwijk, T. M. Proximity effect under nonequilibrium conditions in double-barrier superconducting junctions. *Phys. C Supercond.* **210**, 21–34 (1993).
5. Lambert, C. J. & Raimondi, R. Phase-coherent transport in hybrid superconducting nanostructures. *J. Phys. Condens. Matter* **10**, 901 (1998).
6. Belzig, W., Wilhelm, F. K., Bruder, C., Schön, G. & Zaikin, A. D. Quasiclassical Green's function approach to mesoscopic superconductivity. *Superlattices Microstruct.* **25**, 1251–1288 (1999).
7. Kuprianov, M. Y. & Lukichev, V. F. Influenceof boundary transparency on the criticalcurrent of 'dirty' SS'S structures. *Sov Phys JETP* **94**, 139–149 (1988).
8. Fominov, Ya. V. & Feigel'man, M. V. Superconductive properties of thin dirty superconductor–normal-metal bilayers. *Phys. Rev. B* **63**, 094518 (2001).
9. Kopnin, N. & Kopnin, N. *Theory of nonequilibrium superconductivity*. (Oxford University Press, Oxford, New York, 2001).
10. Pronin, A. V. *et al.* Direct observation of the superconducting energy gap developing in the conductivity spectra of niobium. *Phys. Rev. B* **57**, 14416–14421 (1998).
11. Wakamura, T., Hasegawa, N., Ohnishi, K., Niimi, Y. & Otani, Y. Spin injection into a superconductor with strong spin-orbit coupling. *Phys. Rev. Lett.* **112**, 036602 (2014).
12. Choi, G.-M. & Cahill, D. G. Kerr rotation in Cu, Ag, and Au driven by spin accumulation and spin-orbit coupling. *Phys. Rev. B* **90**, 214432 (2014).
